# *Culicoides* Species Communities Associated with Wild Ruminant Ecosystems in Spain: Tracking the Way to Determine Potential Bridge Vectors for Arboviruses

**DOI:** 10.1371/journal.pone.0141667

**Published:** 2015-10-28

**Authors:** Sandra Talavera, Francesc Muñoz-Muñoz, Mauricio Durán, Marta Verdún, Anna Soler-Membrives, Álvaro Oleaga, Antonio Arenas, Francisco Ruiz-Fons, Rosa Estrada, Nitu Pagès

**Affiliations:** 1 IRTA, Centre de Recerca en Sanitat Animal (CReSA, IRTA- UAB), Campus de la Universitat Autònoma de Barcelona, Bellaterra, Catalonia, Spain; 2 Departament de Biologia Animal, de Biologia Vegetal i d’Ecologia, Universitat Autònoma de Barcelona, Bellaterra, Catalonia, Spain; 3 Health and Biotechnology (SaBio) group, Instituto de Investigación en Recursos Cinegéticos (IREC), Ciudad Real, Castilla la Mancha, Spain; 4 SERPA, Sociedad de Servicios del Principado de Asturias S.A., Gijón, Asturias, Spain; 5 Departamento de Sanidad Animal, Facultad de Veterinaria, Universidad de Córdoba (UCO), Córdoba, Andalucía, Spain; 6 Department of Animal Pathology, Faculty of Veterinary, University of Zaragoza, Zaragoza, Spain; University of Thessaly, GREECE

## Abstract

The genus *Culicoides* Latreille 1809 is a well-known vector for protozoa, filarial worms and, above all, numerous viruses. The Bluetongue virus (BTV) and the recently emerged Schmallenberg virus (SBV) are responsible for important infectious, non-contagious, insect-borne viral diseases found in domestic ruminants and transmitted by *Culicoides* spp. Both of these diseases have been detected in wild ruminants, but their role as reservoirs during the vector-free season still remains relatively unknown. In fact, we tend to ignore the possibility of wild ruminants acting as a source of disease (BTV, SBV) and permitting its reintroduction to domestic ruminants during the following vector season. In this context, a knowledge of the composition of the *Culicoides* species communities that inhabit areas where there are wild ruminants is of major importance as the presence of a vector species is a prerequisite for disease transmission. In this study, samplings were conducted in areas inhabited by different wild ruminant species; samples were taken in both 2009 and 2010, on a monthly basis, during the peak season for midge activity (in summer and autumn). A total of 102,693 specimens of 40 different species of the genus *Culicoides* were trapped; these included major BTV and SBV vector species. The most abundant vector species were *C*. *imicola* and species of the Obsoletus group, which represented 15% and 11% of total numbers of specimens, respectively. At the local scale, the presence of major BTV and SBV vector species in areas with wild ruminants coincided with that of the nearest sentinel farms included in the Spanish Bluetongue Entomological Surveillance Programme, although their relative abundance varied. The data suggest that such species do not exhibit strong host specificity towards either domestic or wild ruminants and that they could consequently play a prominent role as bridge vectors for different pathogens between both types of ruminants. This finding would support the hypothesis that wild ruminants could act as reservoirs for such pathogens, and subsequently be involved in the reintroduction of disease to livestock on neighbouring farms.

## Introduction

Around 1,400 species of biting midges of the genus *Culicoides* have been described in the world [1]; some of these are well known transmitters of protozoa, filarial worms and viruses that affect humans and domestic and/or wild animals [2]. One of the most important of these pathogens is Bluetongue virus (BTV), which is a double stranded RNA virus of the genus *Orbivirus* that produce an infectious, non-contagious disease that affects domestic and wild ruminants [3]. At the global scale, BTV is one of the most economically important diseases transmitted by *Culicoides* in terms of the disruption of both international and domestic trade [4]. Over the last decade, BT has re-emerged in the Mediterranean countries. The spread of this disease was initially associated with the introduction and establishment of the main vector for BTV outbreaks in Africa and Southern Europe, the Afro-Asiatic species *Culicoides imicola* Kieffer, 1913. Once this disease had become established in Southern Europe, BTV-8 unexpectedly appeared in Western and Central Europe in August 2006, where *C*. *imicola* was absent, and where endemic species of *Culicoides* such as *C*. *obsoletus* and *C*. *scoticus* [5], [6, 7, 8], *C*. *dewulfi* [9], *C*. *chiopterus* [10] and *C*. *pulicaris* [11] were pointed to as potential vectors for the disease. *Culicoides* have recently been identified as potential carriers of Schmallenberg virus (SBV), based on both field [12–15] and laboratory [16] studies. The virus produces a disease that affects ruminants and which was first detected in Germany and the Netherlands in the summer and autumn of 2011 [17]. Since then, it has spread throughout almost the whole of Europe and its presence was confirmed in Spain (in March 2012) when it affected sheep and goats in the south of the country [18]. To date, eight species of *Culicoides* have been described as vectors for SBV in Europe: *C*. *obsoletus*, *C*. *scoticus*, *C*. *dewulfi*, *C*. *chiopterus* [12, 13, 14], *C*. *punctatus* [15], *C*. *pulicaris*, *C*. *nubeculosus* and *C*. *imicola* [16]; all of these are considered vectors of BTV except *C*. *punctatus* and *C*. *nubeculosus*.

Seven different species of wild ruminants are present in Spain; the red deer (*Cervus elaphus* Linnaeus, 1758), which is the most abundant species; the fallow deer (*Damma damma* Linnaeus, 1758); the roe deer (*Capreolus capreolus* Linnaeus, 1758); the mouflon (*Ovis aries musimon* Pallas, 1762); the Spanish ibex (*Capra pyrenaica hispanica* Schinz, 1838); the Pyrenean chamois (*Rupicapra pyrenaica* Bonaparte, 1845); and the aoudad or Barbary sheep (*Ammotragus lervia* Pallas, 1777) [19]. The infection of wild ruminants by BTV and SBV has been previously reported and specific antibodies to BTV have been detected in all of the previously listed species in Spain [20–24], and to SBV in several wild ruminant species: red deer, roe deer, fallow deer, European bison, elk, chamois and Pyrenean chamois in other parts of Europe [25–29]. The role played by wild ruminants in relation to the maintenance of disease and its dissemination to domestic ruminants has so far received little attention, although recent studies suggest their involvement in the disemination of BTV and its persistence in Spain [23, 24]. The detection and control of Bluetongue and Schmallenberg in wild ruminants is difficult, particularly as most species are asymptomatic to BTV [30] and SBV [27, 28]; controlling *Culicoides*-borne pathogens that come from wild populations is therefore extremely difficult.

The characteritzation of *Culicoides* midge communities in areas in which wild ruminants are present is important for understanding the role that wild ruminants could play in the dynamics of BTV and SBV. To our knowledge, the composition of *Culicoides* communities in these areas has so far been poorly studied in Europe and deserves greater attention. The main objective of the present study was therefore to characterize *Culicoides* midge communities in forest environments where wild ruminants were present and abundant and to compare such communities with those found close to livestock. To achieve this main goal, the following specific objectives were established: i) to determine the relative abundance of *Culicoides* species within wild ruminant areas, ii) to reveal whether the main vector species present on livestock farms are also present in wild ruminant areas, and whether they could therefore act as bridge vectors between the two types of ruminants, and finally iii) to determine whether some mammalophilic *Culicoides* species (or ones without known host preferences) are absent from livestock farms in areas also inhabited by wild ruminants.

## Materials and Methods

### Sampling

The *Culicoides* specimens identified in the study were trapped in 2009 and 2010, during the main *Culicoides* activity season (from July to November). They were captured on seven Spanish private areas characterized by their distinctive bioclimatic features and wild ruminant communities (Table 1). Data relating to bioclimatic variables and altitude were obtained from the climatic atlas of the Iberian Peninsula [31]; landscape variables were obtained from the Global Environment Monitoring database [32], and the distribution of the different ruminants within Spain was obtained from an atlas of land mammals in Spain [19]. Permanent single trapping sites were established near water sources in each area; these were usually located more than 1 km from the closest livestock farm. Food and water were provided to wild ruminants on a regular basis at Puig la Penya and El Juanar. Three CDC black light traps (John W. Hock Company, Gainesville, FL, USA) were placed at each sampling site and used from dusk to dawn on three consecutive nights, once per month. The CDC traps were employed to ensure results that would be comparable with data from the Spanish Bluetongue National Surveillance Programme (which also used CDC black light traps). Trapped insects were collected in containers containing soapy water and were then stored in 70% ethanol for morphological identification. Access to private land was granted by the respective landowners. Fieldwork did not involve any endangered or protected species.

**Table 1:** Data summary of ecological variables and characterization of the sampling sites [19, 32].

Sampling site	Code	Geographical variables		Bioclimatic variables				Environment near the sampling site		Ruminant	Ruminants in sampling place	*Culicoides*	Landscape
		Latitude	Longitude	A(m)	HT(°C)	LT(°C)	AP(mm)	Domestic ruminants (distance in km)	Water (distance in meters)	(wild—domestic)	(more abundant—less abundant)	% total (both sexes)	
**Proaza**	**1W**	43.203361	-6.055999	349	25	0.0	1,000	Farm (4,5)	wet soil, not water on surface	wild	red deer	5.9	Shrub Cover, closed-open, deciduous
**R.N.C.Boumort**	**2W**	42.201691	1.099684	1,276	25	-7.5	900	Free domestic livestock (1)	Pond (<5)	wild	red deer, fallow deer, roe deer, chamois	0.1	Tree Cover, broadleaved, deciduous, closed
**Puig la Penya**	**3W**	42.307891	2.803300	228	28	2.5	1,000	Farm (2)	Pond (25)	wild	red deer, fallow deer, mouflon	4.4	Tree Cover, needle-leaved, evergreen
**Quintos de Mora**	**4W**	39.383333	-4.100000	718	35	0.0	482	Farm (10)	Seasonal stream (<5)	wild	red deer, fallow deer, roe deer	27.6	Tree Cover, needle-leaved, evergreen
**La Morera**	**5W**	38.911372	-4.260120	707	35	2.5	500	Farm (4)	Pond (5)	wild	red deer, mouflon, aoudad	6.5	Shrub Cover, closed-open, evergreen
**El Juanar**	**6W**	36.569647	-4.890657	870	28	2.5–5.0	850	Farm (5)	Cement trough (<5)	wild	Spanish ibex	0.9	Tree Cover, needle-leaved, evergreen
**La Almoraima**	**7W**	36.289592	-5.431023	45	31	7.5	955	Farm (4)	wet soil, not water on surface	wild	red deer, fallow deer, roe deer, mouflon	54.6	Cultivated and managed areas
**Tineo**	**1D**	43.194797	-6.251201	673	25	0.0	1,000	-	-	domestic	cow	-	Cultivated and managed areas
**Aramunt**	**2D**	42.206277	0.987546	559	23	-2.5	700	-	-	domestic	sheep	-	Cultivated and managed areas
**Vilanova de la Muga**	**3D**	42.303944	3.031915	19	25	2.5	800	-	-	domestic	sheep	-	Cultivated and managed areas
**Piedrabuena**	**4D**	39.147973	-4.101201	584	35	0.0	400	-	-	domestic	cow	-	Cultivated and managed areas
**Navacerrada**	**5D**	38.453675	-4.260202	614	37.5	0.0	500	-	-	domestic	cow, sheep, goat	-	Cultivated and managed areas
**Mijas**	**6D**	36.311594	-4.421226	428	32.5	5.0	700	-	-	domestic	sheep, goat	-	Cultivated and managed areas
**Castellar de la Frontera **	**7D**	36.191199	-5.270036	47	32.5	5.0–7.5	1,000	-	-	domestic	sheep, goat	-	Cultivated and managed areas

A, altitude; AP, annual precipitation; LT, mean low temperature of the coldest month; HT, mean high temperature of the warmest month [31].

In order to compare the composition of the *Culicoides* vector species between areas occupied by domestic and wild ruminants, contemporary data were obtained from the Spanish Bluetongue National Surveillance Programme relating to seven livestock farms (Table 2). These were the farms located closest to the seven study sites with wild ruminants (which were less than 60 km apart). Although the data from the Spanish Bluetongue National Surveillance Programme only included data for known BT vector species, data for all the trapped *Culicoides* species were also available for farms at Vilanova de la Muga and Aramunt (which were included in communitiy analyses).

**Table 2:** Vector species or species group abundance (n° midge/night/trap) at wild and domestic ruminants sampling sites.

Sampling Site	Site	Ruminant site	*C*. *imicola*	Obsoletus group	Pulicaris group	N (n°midges/night/trap)	% N
Tineo	1D	Domestic	0.00	27.95	1.70	29.65	0.29
Aramunt	2D	Domestic	0.00	1,081.15	71.80	1,152.95	11.42
Vilanova de la Muga	3D	Domestic	0.00	15.70	1.15	16.85	0.17
Piedrabuena	4D	Domestic	155.00	2.00	0.00	157.00	1.56
Navacerrada	5D	Domestic	258.75	0.60	0.00	259.35	2.57
Mijas	6D	Domestic	418.17	2.75	0.00	420.92	4.17
Castellar de la Frontera	7D	Domestic	52.32	0.00	0.00	52.32	0.52
Total domestic ruminant sites			884.24	1,130.15	74.65	2,089.04	20.70
Proaza	1W	Wild	0.00	1,186.59	55.60	1,242.19	12.31
R.N.C. Boumort	2W	Wild	0.00	7.31	5.97	13.28	0.13
Puig la Penya	3W	Wild	0.00	546.95	83.63	630.58	6.25
Quintos de Mora	4W	Wild	14.31	212.95	123.27	350.53	3.47
La Morera	5W	Wild	41.62	0.00	5.97	47.59	0.47
El Juanar	6W	Wild	35.31	133.95	109.94	279.20	2.77
La Almoraima	7W	Wild	5,196.61	133.28	110.29	5,440.18	53.90
Total wild ruminant sites			5,287.85	2,221.03	494.67	8,003.55	79.30
						10,092.59	100.00

### Morphological and molecular identification

*Culicoides* midges were first identified, under a stereomicroscope (Nikon SMZ), at the species or species-group level, according to their wing pattern morphology [33] (S1 Table). In addition, females were separated by the gonodotrophic status following the categorization performed by Dyce [34]. In order to perform community analyses at wild ruminant sites, an accurate morphological identification was later performed for all the species cited in the manuscript on dissected individuals slide-mounted in Canada balsam (for at least one individual of each sex). The slides were examined with a Nikon Eclipse E200 light microscope using the main taxonomic keys for Palearctic *Culicoides* [35–39]. It is difficult to separate *C*. *obsoletus* and *C*. *scoticus* females using traditional morphological techniques [40]. In order to confirm the presence of Obsoletus group females, precise identification of 73 females of the Obsoletus group was performed by means of PCR according to the procedures described in [41 and 42].

*Culicoides* species were classified into ornithophilic and mammalophilic according to their feeding habits, based on morphological analysis of main sensory structures such as antennae and palps, ([43] and references therein). Species with sensilla coeloconica (SC) on 8 or more antennal flagellomers, SC on antennal flagellomers 4–10 and 1 large maxillary palp sensory pit, were categorised as ornitophilic. Those with SC on 6 or fewer antennal flagellomers, without SC on antennal flagellomers 4–10 and 1 or more smaller maxillary palp sensory pits were categorised as mammalophilic. Species that did not fit into either of these two categories were categorised as indefinite or unknown.

The different specimens that were deposited, at the CReSA collection were cited using the following abbreviation: INIA-CReSA.

### Statistical analyses

For the *Culicoides* community analyses, a presence/absence dataset of all the vector and non-vector species was established, at a species level, for the seven wild ruminant sites and the two domestic ruminant sites (Aramunt and Vilanova de la Muga). The *Culicoides* species richness (number of species per site) was calculated. Similarities between different *Culicoides* communities were determined using the Bray-Curtis (BC) similarity index [44]. Multivariate non-metric multidimensional scaling (nMDS) was used to asses the relationships between the different *Culicoides* communities at all the different sites. MDS allows visualizing the degree of similariy between the samples in a data matrix by displaying the information contained in a distance/similarity matrix. Data were analyzed using a one-way analysis of similariy (ANOSIM) to test for differences between the presence of domestic or wild ruminant and between neighbouring landscapes. This procedure generates an *R* statistic that quantifies the degree of discrimination between sites and a *p* value that indicates the significance of the differences observed. The R statistic ranges from 0 to 1 and is approximately zero if the null hypothesis is true: when the similarities within sites tend on average to be the same as those between different sites [45]. The projection of vectors in the nMDS ordination finds the directions in the ordination space towards which the environmental vectors change most rapidly and to which they have maximal correlations with the ordination configuration. Then, vectors (arrows) in the nMDS plot represent explanatory environmental variables (bioclimatic variables and altitude, see Table 1) and are proportional in length to their importance. Similarity profile analysis (SIMPROF) was also carried out to statistically detect structuring in *Culicoides* communities. SIMPROF examines null hypothesis by testing whether the similarities observed in the data are larger or smaller than those that could be expected due to chance. A two-way cluster analysis was performed factoring in both sampling sites and species based on BC similarity index of presence/absence data. Two-way cluster analysis independently groups sample sites and species and combines them in a single diagram to allow the observation of associations between different groups of sample units and species.

The abundance data available for relevant vector species or species groups for the 14 different localities were used to test for differences between wild and domestic ruminant sites. For comparative analyses, the data were transformed into n° midges/trap/night because the trapping effort used in the current work was different from the one used by the Spanish Bluetongue National Surveillance Programme. Prior to analysis, the data matrix containing the abundance of vector species per site was square root transformed to reduce the importance of extreme values [44]. Similarities between sites were determined using the BC similariy index and visualized by nMDS. ANOSIM was carried out to test whether the composition of the *Culicoides* community significantly differed according to the type of ruminant species (domestic or wild) considered.

All the multivariate analyses were performed using the Primer 7 software package [46].

## Results

A total of 102,693 specimens of the genus *Culicoides* were trapped during the study period (S1 Table). Of them, 20,970 (20%) were males and 81,723 (80%) were females, with 79.75% being parous and 0.25% blood engorged females [34]. The specimens were assigned to one of 40 different species (Table 3) without any new species being cited for the Iberian Peninsula with respect to the latest taxonomic catalog published by Alarcón-Elbal and Lucientes [47].

**Table 3:** Distribution, morphological features and host-feeding preferences of all the identified species of *Culicoides*.

	Distribution found									Antenna (Sensilla coeloconica)		Maxillary palp (Sensory pit)		
Species	1W	2W	3W	4W	5W	6W	7W	2D	3D	AF with SC	presence/absence AF 4–10	number	size	Host preference
*Culicoides alazanicus* Dzhafarov, 1961	●		●		●				●	13	presence	1	large	birds
*Culicoides (Oecacta) brunnicans* Edwards, 1939					●					9	presence	2	small	indefinite
*Culicoides cataneii* Clastrier, 1957		●	●	●	●	●	●			12–13	presence	1	large	birds
*Culicoides (Beltranmyia) circumscriptus* Kieffer, 1918		●	●	●	●	●	●	●	●	12	presence	1	large	birds
*Culicoides coluzzii* Callot, Kremer & Bailly–Choumara, 1970	●			●	●		●			8	presence	1	large	birds
*Culicoides derisor* Callot & Kremer, 1965			●							6	absence	1	small	mammals
*Culicoides (Avaritia) dewulfi* Goetghebuer, 1936	●									6	absence	1	small	mammals
*Culicoides (Culicoides) fagineus* Edwards, 1939	●			●	●	●		●	●	6	absence	various	small	mammals
*Culicoides festivipennis* Kieffer, 1914	●	●	●	●	●		●			13	presence	1	large	birds
*Culicoides (Culicoides) flavipulicaris* Dzhafarov, 1964								●	●	6	absence	various	small	mammals
*Culicoides furcillatus* Callot, Kremer & Paradis, 1962	●									6	absence	1	small	mammals
*Culicoides gejgelensis* Dzhafarov, 1964		●	●	●		●	●			12–13	presence	1	large	birds
*Culicoides griseidorsum* Kieffer, 1918									●	12	presence	1–2	large	birds
*Culicoides haranti* Rioux, Descous & Pech, 1959			●	●	●					11–13	presence	1	large	birds
*Culicoides heteroclitus* Kremer & Callot, 1965				●	●		●		●	12–13	presence	1	large	birds
*Culicoides (Avaritia) imicola* Kieffer, 1913				●	●	●	●			6	absence	1	small	mammals
*Culicoides (Culicoides) impunctatus* Goetghebuer, 1920	●							●		5–6	absence	various	small	mammals
*Culicoides jumineri* Callot & Kremer, 1969				●						9	presence	1	large	birds
*Culicoides kibunensis* Tokunaga, 1937		●								13	presence	1	large	birds
*Culicoides kurensis* Dzhafarov, 1960			●		●		●			9	presence	1	large	birds
*Culicoides lailae* Khalaf, 1961								●		12	presence	1	large	birds
*Culicoides lupicaris* Downes & Kettle, 1952	●		●							6	absence	various	small	mammals
*Culicoides marcleti* Callot, Kremer & Basset, 1968					●		●			8	presence	1	large	birds
*Culicoides maritimus* Kieffer, 1924			●				●			13	presence	1	large	birds
*Culicoides (Culicoides) newsteadi* Austen, 1921			●	●	●		●	●	●	6	absence	various	small	mammals
*Culicoides (Avaritia) obsoletus* (Meigen, 1818)	●	●	●	●		●	●	●	●	6	absence	1	small	mammals
*Culicoides odiatus* Austen, 1921				●			●			12	presence	1	large	birds
*Culicoides paolae* Boorman, Mellor & Scaramozzino, 1996				●		●	●			13	presence	1	large	birds
*Culicoides (Monoculicoides) parroti* Kieffer, 1922		●	●	●	●		●	●		4	presence	1	small	indefinite
*Culicoides pictipennis* (Staeger, 1839)							●			13	presence	1	large	birds
*Culicoides poperinghensis* Goetghebuer, 1953					●		●			6	absence	1	small	mammals
*Culicoides pseudopallidus* Khalaf, 1961			●		●		●			13	presence	1	large	birds
*Culicoides (Culicoides) pulicaris* (Linnaeus, 1758)		●	●	●	●	●	●	●	●	6	absence	various	small	mammals
*Culicoides (Culicoides) punctatus* (Meigen, 1804)	●	●	●	●	●	●	●	●	●	6	absence	various	small	mammals
*Culicoides (Monoculicoides) puncticollis* (Becker, 1903)				●	●		●			4	presence	1–2	small	indefinite
*Culicoides (Pontoculicoides) saevus* Kieffer, 1922		●		●						5	presence	1	large	indefinite
*Culicoides (Oecacta) sahariensis* Kieffer, 1923			●	●	●	●	●	●		6	presence	1	large	indefinite
*Culicoides (Avaritia) scoticus* Downes & Kettle, 1952	●	●	●	●		●	●	●	●	6	absence	1	small	mammals
*Culicoides (Wirthomyia) segnis* Campbell & Pelham-Clinton, 1960							●			12	presence	1	large	birds
*Culicoides shaklawensis* Khalaf, 1957				●			●			6	absence	1	large	indefinite
*Culicoides (Culicoides) subfagineus* Delécolle & Ortega, 1998				●	●		●			6	absence	various	small	mammals
*Culicoides submaritimus* Dzhafarov, 1962									●	12	presence	1	large	birds
*Culicoides univittatus* Vimmer, 1932				●	●	●	●	●	●	13	presence	1	large	birds
*Culicoides yemenensis* Boorman, 1989				●	●		●			8	presence	1	large	birds
**Species richness**	11	11	18	25	23	12	28	13	13					

1W, Proaza; 2W, R.N.C.Boumort; 3W, Puig la Penya; 4W, Quintos de Mora; 5W, La Morera; 6W, El Juanar; 7W, La Almoraima; 2D, Aramunt; 3D, Vilanova de la Muga. AF, antennal flagellomer; SC, sensilla coeloconica; SP, sensory pit.

**Fig 1:**
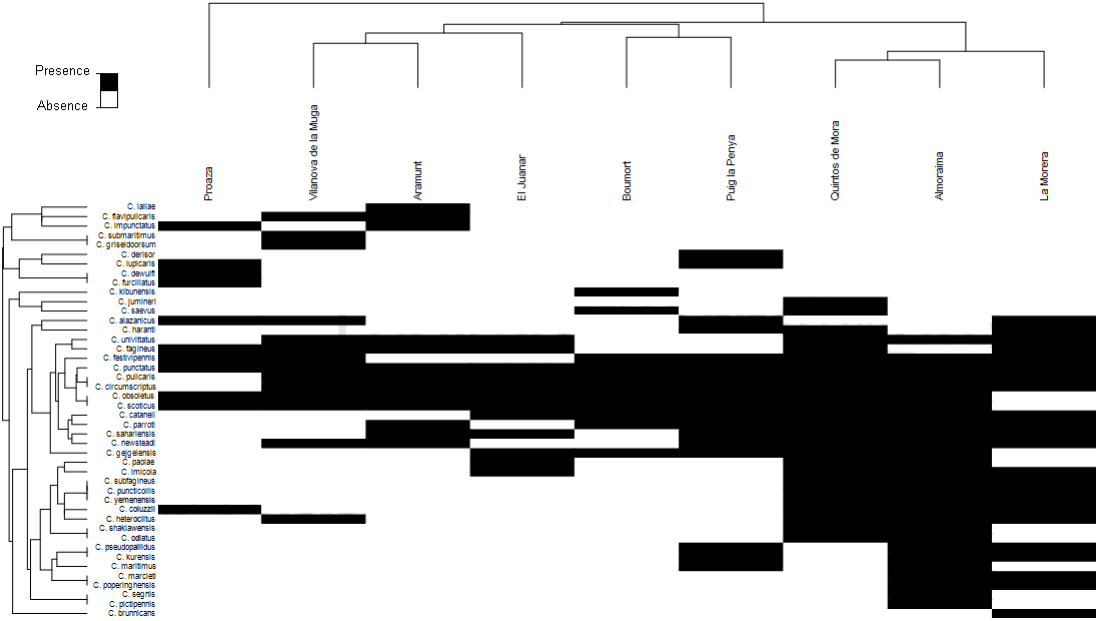
Two-way cluster based on the Bray-Curtis similarity analysis of presence-absence data between *Culicoides* species and localities analyzed.

**Fig 2:**
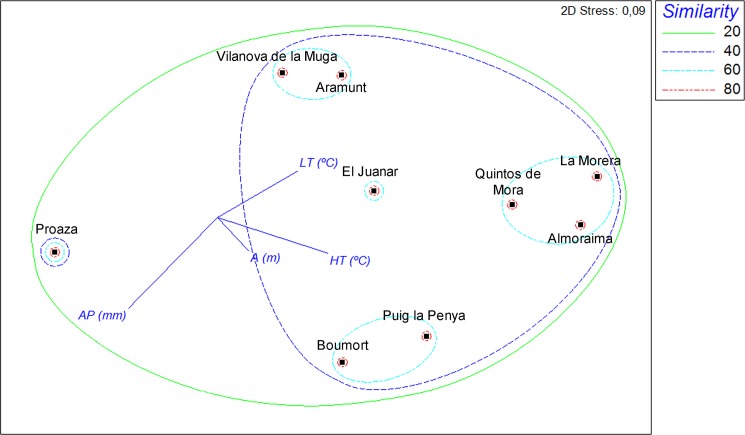
Non-metric multidimensional scaling (MDS) ordination of Bray-Curtis similarity matrix for *Culicoides* communities based on presence-absence data.

Analyzing the richness of species in each of the areas studied, up to 28 species were detected in La Almoraima and at least of 11 species in R.N.C. Boumort and Proaza (Fig 1, Table 3). The mean number of species per site was greater at wild (18.3) than at domestic (13) ruminant sites. Fig 2 shows the nMDS plot for species composition and, according to the SIMPROF tests (*p*<0.05), several groups can be separated. Three groups were detected at similarity levels of 60%: Vilanova de la Muga and Aramunt were grouped together, R.N.C. Boumort and Puig la Penya formed another group, and Quintos de Mora, La Morera and La Almoraima were also considered to have similar *Culicoides* communities. At this similarity level, El Juanar was judged to constitute a separate group of its own. Proaza was very different the other groups, with less than 40% similarity. The *Culicoides* communities associated with domestic and wild ruminant sites were similar (ANOSIM, global *R* = 0.175, *p* = 0.250), indicating that the presence of one ruminant type ore another did not affect the species composition (presence/absence data). The ANOSIM results also showed that landscape did not have a significant influence on the observed variations in species composition (global *R* = 0.233, *p* = 0.207). The nMDS (Fig 2) revealed annual precipitation to be the factor that most explained the community patterns, according to its vector length and direction. The vector for annual precipitation divided localities in two dimensions, i.e. those located low and left-hand side of the graph, which had higher levels of annual precipitation, and those situated high and to the right-hand side. High and low temperatures also correlated with the ordination in a left-right dimension. The Pearson's correlation coefficient between the altitude and the given ordination axis is <0.2, there was therefore no relationship between the ordination of the sites and their altitude.

ANOSIM showed no significant differences neither between the type of ruminant (wild and domestic) sites nor among landscape features. Environmental variables appear as vectors that indicate relative correlation with MDS axes: LT (mean low temperature of the coldest month, in °C), HT (mean high temperature of the warmest month, in °C), AP (annual precipitation, in mm) and altitude (in meters).

Regarding the raw abundance (S1 Table), the 41.9% of specimens belonged to species with few or no spots on their wings. The rest of species, with spots on the wings, showed both a heterogeneous distribution and abundance. *Culicoides imicola* represented 15.4% of the overall total, 98.3% of which were found in La Almoraima. *Culicoides festivipennis* constituted 15.7% of the total captures, 79.7% of which were caught in Quintos de Mora. The Obsoletus group represented 6.6% of the captures, 54% of which were from Proaza and *C*. *circumscriptus* 9.4%, 70.8% of which were found in Quintos de Mora. Other less frequent detected species included: the Pulicaris group 1.5%, *C*. *newsteadi* 2.5%, the Similis group 3.4%, *C*. *punctatus* 1.2%, *C*. *parroti* also 1.2% and a final group including *C*. *impunctatus*, *C*. *puncticollis*, *C*. *shaklawensis*, *C*. *paolae*, the Odibilis group, *C*. *alazanicus*, the Fagineus group and the Sphagnumensis group 1.2%

**Fig 3:**
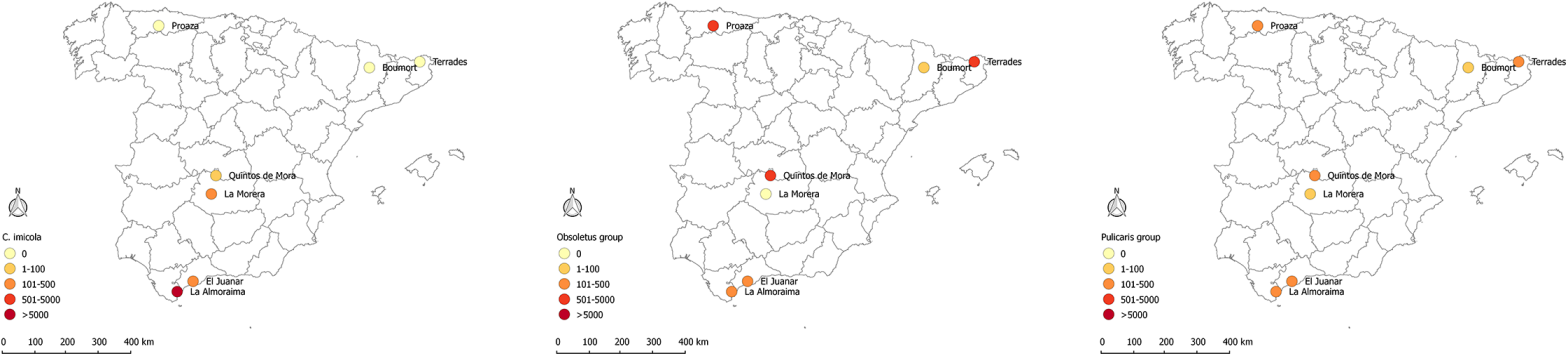
Map showing the abundance of the main vector species of BTV and SBV in the sampling sites.

**Fig 4:**
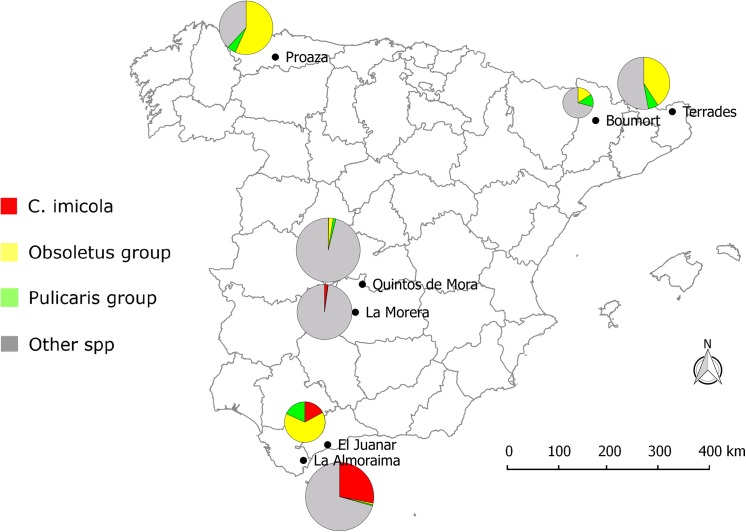
Map showing the relative abundance of the main vector species of BTV and SBV in the sampling sites.

**Fig 5:**
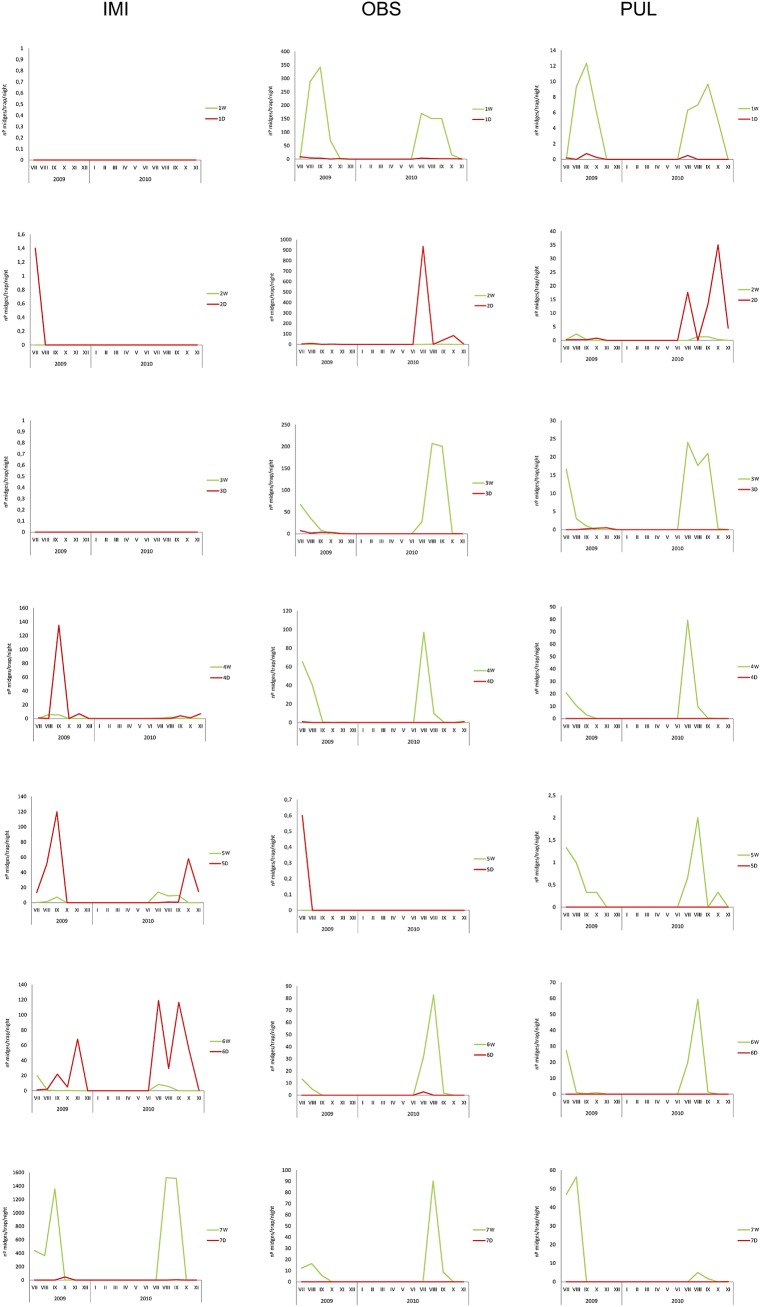
Monthly n°midges/trap/night of the main vector species of BTV and SBV from July to December 2009 and from January to November 2010 at each wild and domestic ruminant sampling site. 1W, Proaza; 2W, R.N.C. Boumort; 3W, Puig la Penya; 4W, Quintos de Mora; 5W, La Morera; 6W, El Juanar; 7W, La Almoraima; 1D, Tineo; 2D, Aramunt; 3D, Vilanova de la Muga; 4D, Piedrabuena; 5D, Navacerrada; 6D, Mijas; 7D, Castellar de la Frontera.

At least one of the epidemiologically relevant species (the known BT vectors: *C*. *imicola*, and species of the Obsoletus and Pulicaris groups) was present at each sampling site (Table 2, Figs 3 and 4). The nMDS perfomed on the fourth root transformed abundances of epidemiologically relevant species based of the BC distance showed no significant differences between domestic and wild ruminant sites (ANOSIM, global *R* = 0.119, *p* = 0.146). *Culicoides imicola* was present at the four southern and central sites (4D-7D, 4W-7W), but absent from the other three north sites (1D-3D, 1W-3W, 2W; Table 2); the only exception was 2D, where a few females where unexpectedly trapped in July 2009 (Fig 5). This species was more abundant at livestock farms (4D-6D) than at corresponding areas with wild ruminants (4W-6D), although the pattern was reversed for site (7D and 7W; Table 2). During the study period (July 2009 to November 2010) *C*. *imicola* displayed a similar pattern in the southern and central sites, being detected from July to October (Fig 5). Species belonging to the Obsoletus group were found at all the sites except two (7D, 5W), being their captures anechdotically at 2W, 4D-6D (Table 2). This species was more abundant in areas where wild ruminants were present than on livestock farms. However, the geographic region 2 (2W-2D) was an exception to that, being the captures at 2D the most abundant of all sites, while anechdotically at 2W (Table 2). At northern sites their abundance was greater than in central and southern ones (Table 2). Species of the Obsoletus group, where present, displayed a similar activity pattern, being detected from June to November (Fig 5).

Species belonging to the Pulicaris group were trapped at all of the wild ruminant sites in medium levels of abundance (Table 2, Fig 3), while at livestock farms these species was either absent (4D-7D) or very scarce (1D-3D) with the exception of site 2D (Table 2). The Pulicaris group was active from June to November (Fig 5). When grouping sites according to ruminant type (wild or domestic), it was noted that the abundance of vector species was much higher at sites with wild ruminants (79.3%) than at livestock farms (20.7%) (Table 2).

In the case of the inferred feeding habits of the different species of *Culicoides* at wild ruminant sites, 50% of the species found in our study were classified as bird-feeders (ornitophilic), 35% as mammal-feeders (mammalophilic) and 15% as indefinite, or with unclear host preference (Table 3). At livestock farms 2D and 3D, 41.2% of the species were ornitophilic, 47.0% were mammalophilic and 12.8% were indefinite (Table 3).

## Discussion

Few studies have been performed on *Culicoides* populations associated to natural areas with wild ruminants, and most of them are focused on parasites affecting wild bird populations [48–52]. The authors only found two studies that had been carried out in areas with wild ruminants, these had been conducted in Spain [53] and Nigeria [54].

Although a wide range of variation in the number of species present at different wild ruminant sites was detected, the results obtained in the present study showed that areas inhabited by wild ruminants tend to be very rich in *Culicoides* species (Table 3). It should also be noted that these values may have been underestimated, as diurnal species are not usually captured by CDC blacklight traps [55]. While Proaza (1W), R.N.C. Boumort (2W) and El Juanar (6W) had a relatively low number of species (S = 11–12), Quintos de Mora (4W), La Almoraima (7W) and La Morera (5W) had many (S = 22–28) (Table 3). Despite the variety of species identified it should be noted that all of the species detected in the present study had previously been recorded at livestock farms [47]. As can be seen from the MDS plot, Quintos de Mora, La Almoraima and La Morera grouped together (Fig 2). The two-way cluster suggests a group of species being exclusive from these southern and central localities, *C*. *subfagineus*, *C*. *yemenensis* and *C*. *puncticollis*. Proaza did not clustered with any other site, and was characterized by the presence of *C*. *dewulfi* and *C*. *furcillatus* and the absence of two otherwise widespread species *C*. *pulicaris* and *C*. *circmuscriptus*. The remaining sites are mostly characterized by the presence of the most common species (*C*. *obsoletus*, *C*. *scoticus*, *C*. *festivipennis*, *C*. *pulicaris* and *C*. *circumscriptus*) and the absence of the previously commented species. Although both of the two farms included in this analysis (Vilanova de la Muga and Aramunt) grouped together, the type of ruminant (wild vs domestic) had no influence on this grouping (Figs 1 and 2). In general, annual precipitation and the mean high temperature for the warmest month seemed to be the bioclimatic variables that most affected groupings, while the mean low temperature in the coldest month and altitude seemed to have a weak effect [56].

The distribution and relative abundance of epidemiologically relevant mammalophilic species (*C*. *imicola* and species belonging to the Obsoletus and Puliaris groups) at the different study sites (with wild ruminants) matched the known geographic pattern inferred from data obtained from the Spanish Bluetongue Entomological Surveillance Program (Table 2). *Culicoides imicola*, which is the main BTV vector in the Mediterranean Basin [3,57,58], was detected in the 4 southern and central areas, i.e. the warmest parts of Spain, but absent from the northern ones (Table 2, Figs 3 and 4). The large scale distribution pattern seems to be strongly influenced by the requirements of the species for high summer temperatures and dry summer conditions [59]. *Culicoides imicola* was more abundant at livestock farms than at natural areas with wild ruminants, with the exception of site 7W-7D (Table 2). Neverthless, the activity patterns of the different species were similar at the central and southern sites. Interestingly, in areas with wild ruminants, *C*. *imicola* was active from July to September, whereas at the central and southern livestock farms, its activity continued until November (Fig 5). The Obsoletus group was present in all of the areas except in La Morera. However, it abundances were much greater at the northern than central or southern sites (with the exception of site 2D; Table 2). The activity pattern was homogeneous for all the natural areas with both wild ruminants and livestock farms, with the activity period being from July to October (and rarely until November, Fig 5). This distribution has been explained by the fact that species belonging to Obsoletus group requires areas with relatively low annual average T^a^ and high soil moisture [60]. These results are in line with Calvete et al. [59], who described a similar latitudinal abundance pattern for livestock farms on the Iberian Peninsula. While *C*. *imicola* predominated in the warmest zones, species from the Obsoletus group predominated in those with relatively low mean annual temperatures. Although being located in the south, El Juanar had a species composition and relative vector abundances similar to northen localities. This pattern could have been influenced by bioclimatic values (Table 1), but also by other factors such as the abundance of suitable hosts and the presence of appropriate breeding sites [61]. In contrast to what was observed for *C*. *imicola* and the Obsoletus group, the species belonging to the Pulicaris group were captured in all of the different natural areas with low to medium abundance values (Fig 2). Interestingly, such a pattern was not found for livestock farms, where the Pulicaris group was absent from all the central and southern farms (Table 2). At the sites in central Spain, none of the mammalophilic species stood out for its abundance; as a result, *C*. *imicola*, *C*. *punctatus* and species belonging to the Obsoletus and Pulicaris groups were trapped in similar (low) quantities (Table 2).

Important differences in the relative abundance of males and females were detected at the wild ruminant sites (S1 Table). The percentage of parous females captured was high (80%). Parous females are those that have completed at least one gonodotrophic cycle and which are already bloodfed and able to be infected if fed on a viraemic host. The active dispersal of adult midges belonging to the genus *Culicoides* is usually quite short, usually being limited to a few hundred metres from their breeding sites and at most to 2–3 km/day [62, 2], and only under very specific temperature, wind and humidity conditions they can become displaced over larger distances by wind [63, 64]. Since the livestock farms closest to the study sampling sites were at distances of between 1 and 10 km (Table 1), it could be assumed that most of the captured females that already had bloodmeal would have biten feral fauna. Regarding the feeding habits of the different species of *Culicoides*, it should be noted that the classification used in this work (Table 3) was based on morphological aspects [43] that were similar to those used in works that used molecular approaches to identify midge bloodmeals [65, 66]. In general, when comparing livestock farms, in natural areas with wild ruminants, it was possible to detect an increase in the relative abundance of ornitophilic species, such as *C*. *circumscriptus* and *C*. *festivipennis* (with these being most abundant at Quintos de Mora), and species with an unclear host preference, such as those belonging to the Similis group and *C*. *parroti*. Such an increase in abundance could be attributable to the greater variety of hosts and lower ruminant availability (density) to feed on in such natural areas [67, 68]. Until now, *Culicoides* species with ornitophilic and indefinite feeding habits had not been considered epidemiologically important for Bluetongue or Schmallenberg diseases. However, some studies have recently shown that *Culicoides* can be opportunists feeders, with species previously considered as ornitophilic or indefinite feeders have been detected feeding on mammals [69, 52, 70]. The fact that they represent 65% of the *Culicoides* caught in the wild ruminant areas, highlights the importance of conducting further studies to obtain more precise information about the feeding patterns of ornitophilic species and those with unclear feeding habits.

With regard the specific objectives of this study, our results showed: i) the composition of *Culicoides* species did not depend on the ruminant type present, ii) the main vector species for BTV and SBV present on the livestock farms were also present in neighbouring natural areas with wild ruminants, which would support their putative role as bridge vectors for the transmission of arboviruses between domestic and wild ruminants (in addition to their recognised role as epizootic vectors) and iii) the presence of non-vector *Culicoides* species in areas with wild ruminants that had previously been found in association with domestic ruminants, suggesting an irrelevant role in the maintenance of *Culicoides* transmitted arboviruses to wild ruminants in the region. Ornitophilic and indefinite species were more abundant in areas with wild ruminants than in those with livestock farms, with the abundance of mammalophilic species being reduced.

Overall, the present study would support the hypothesis that wild ruminant communities could serve as arbovirus reservoirs for *Culicoides* transmitted arboviruses. Wild ruminants are susceptible to various *Culicoides* transmited viral diseases and our data confirmed that they are in close contact with major *Culicoides* vector species. Well known *Culicoides* vector species (*C*. *imicola* and Obsoletus group) could act as bridge vectors and circulate pathogens at the interface between wild and domestic ruminant communities. Based on this hypothesis, the bypass of the pathogen among wild/domestic communities mediated by *Culicoides* bridge vectors (*C*. *imicola* and Obsoletus group) would facilitate the interseasonal BTV and SBV reintroduction among domestic ruminants. To further support the hypothesis, future studies will be needed to determine the bloodfeeding preferences of *Culicoides* in areas where wild ruminants are present.

## Supporting Information

S1 Table Species or species group abundance at each wild ruminanats sampling sites. [71]. P, parous; N, nuliparous [34].
(XLSX)
